# Training Classifiers with Shadow Features for Sensor-Based Human Activity Recognition

**DOI:** 10.3390/s17030476

**Published:** 2017-02-27

**Authors:** Simon Fong, Wei Song, Kyungeun Cho, Raymond Wong, Kelvin K. L. Wong

**Affiliations:** 1 Department of Computer and Information Science, University of Macau, Taipa 999078, Macau, China; 2 Department of Digital Media Technology, North China University of Technology, Beijing 100144, China; sw@ncut.edu.cn; 3 Department of Multimedia Engineering, Dongguk University, Seoul 04620, Korea; cke@dongguk.edu; 4 School of Computer Science and Engineering, University of New South Wales, Sydney 2052, NSW, Australia; wong@cse.unsw.edu.au; 5 School of Medicine, Western Sydney University, Sydney 2560, NSW, Australia

**Keywords:** feature selection, supervised learning, classification, human activity recognition

## Abstract

In this paper, a novel training/testing process for building/using a classification model based on human activity recognition (HAR) is proposed. Traditionally, HAR has been accomplished by a classifier that learns the activities of a person by training with skeletal data obtained from a motion sensor, such as Microsoft Kinect. These skeletal data are the spatial coordinates (*x*, *y*, *z*) of different parts of the human body. The numeric information forms time series, temporal records of movement sequences that can be used for training a classifier. In addition to the spatial features that describe current positions in the skeletal data, new features called ‘shadow features’ are used to improve the supervised learning efficacy of the classifier. Shadow features are inferred from the dynamics of body movements, and thereby modelling the underlying momentum of the performed activities. They provide extra dimensions of information for characterising activities in the classification process, and thereby significantly improve the classification accuracy. Two cases of HAR are tested using a classification model trained with shadow features: one is by using wearable sensor and the other is by a Kinect-based remote sensor. Our experiments can demonstrate the advantages of the new method, which will have an impact on human activity detection research.

## 1. Introduction

Human activity recognition (HAR) is a branch of machine learning that trains a classification model using historical activity records to recognise unseen activities. HAR has wide application, ranging from abnormal behaviour detection in security surveillance [1] and posture monitoring in tele-rehabilitation [2], to hand gesture recognition in augmented-reality [3], and virtual-reality [4] systems, etc.

One type of HAR is based on motion sensor data from which the HAR system tries to infer activity patterns for prediction and classification. The sensor data arrive in the form of continuous and sequential values, similar to time series. Time series, such as those collected from an accelerometer, are usually multivariate, comprising tri-axial spatial information, known in its simplest form as ‘three features’ (*x*, *y*, *z*). These three features, together with the time-stamp information in the time series, represent the temporal-spatial displacement of a body part in motion. More complicated time series may contain extra information, such as velocity, acceleration, rotational angle, and relative distance to a reference point.

**Figure 1:**
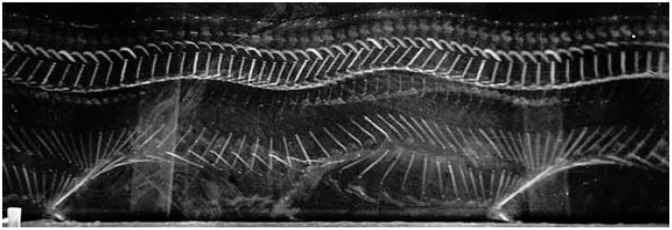
Cinematographic movement of marching soldier *(image courtesy of Motion Studies predating Taylorism, Images produced by Etienne-Jules Marey*).

In this paper, a new type of feature, which we term as a ‘shadow feature’ is presented. The concept of shadow features is inspired by Etienne-Jules Marey (1830–1904), who instigated chronophotography, or cinematographic movement recording [5], as a fundamental technique of capturing motion pictures. His lifetime passion ‘to see the invisible’ motivated him towards a single goal: recording all existing movement, produced by humans or anything else, on a recordable surface. In alignment with Etienne-Jules’ motivation, shadow features are derived from the dynamics of human body movements. The shadow feature values are subtly approximated by the underlying motion resulting from performing activities. For example, in Figure 1, the motion of a marching solider can be seen in a chronophotograph as longitudinal curves in a time series.

The shadow feature approximation is carried out quickly by a time-series smoothing technique coupled with the Hurst factor for fast-processing (described in detail in Section 3). Unlike the other feature transformation techniques that have been previously reported in the literature, the shadow features method does not transform and replace the original features; instead, it creates a new set of motion dynamic data that augments the original spatial data. Hence, the term ‘shadow’ arises here.

Shadow features offer extra dimensions of information for characterising activities in the HAR classification process. The extra feature information gives insight into the motion of the body in supervised learning. The classification algorithm tries to learn and induce relationship mapping between the input feature values and the target classes. It can better learn about, and recognise, the activities when provided with the motion information in addition to spatial coordinate data. Extra maps between the motion information and the target activities can be built to complement the existing mapping based on spatial information.

Not only is the shadow features method designed to improve classification accuracy, its advantage over peer techniques pertains to its incremental nature. Simple and lightweight in computation, this method can generate extra feature values on the fly, making it suitable for data mining time series data, such as sensor data used in the classification of HAR. This quality is important in the HAR environment, where the incoming data are fast-moving in nature and the sensing data can potentially amount to infinity. Shadow features can be incrementally extended such that the classification model learns incrementally as data streams in. In contrast, feature transformation techniques, such as wavelet transformation [6] and statistical transformation techniques [7], require all of the data to be available to calculate the new feature values. This induces heavy latency in HAR because the full dataset must be reloaded into the classification model to rebuild (instead of refresh) the whole classifier all over again. 

The contribution of this paper is two-fold, and addresses the dual goals of recognition accuracy and speed. A classification model that capitalises on the shadow features method is proposed for better classification accuracy in HAR. The new model is tested in two popular groups of traditional supervised learning algorithms (which learn over a full batch of data) and lazy incremental learning algorithms (which quickly update the model upon the arrival of new data). This research is a step towards using the shadow features method in data stream mining models with incremental feature generation for HAR.

Our paper is organised as follows: Section 2 reviews related work and summarises mainstream feature selection and transformation techniques for HAR; Section 3 presents our proposed classification model incorporating the new shadow features method; experiments comparing the original and new methods using datasets from an accelerometer and Kinect motion camera (Microsoft Corporation, Redmond, WA, USA) are reported in Section 4; and, finally, Section 5 concludes the paper.

## 2. Related Work

HAR is a multi-disciplinary technology with research fields in software and hardware related to computer vision, human–computer interactions, machine learning, time series data mining, data transformation, and system design. In this brief review, we focus on time series transformation: how time series are extracted, transformed, and represented from raw sensor data, and the effect on recognition accuracy.

Features that can be extracted from raw sensor data are loosely categorised into remote features and local features. Remote features are data from a sensor at a fixed position (such as a video camera, Kinect motion sensor, and ultrasound sensor) that captures images of a moving subject from some distance away. Local features are data collected from wearable sensors (for example), which ‘tag along’ with the subject on the go. Traditionally, discrete Fourier transformation (DFT) [8] is applied to a sequence of image frames to convert the image intensity variation spatially from the temporal domain to the frequency domain. DFT extracts information from the information contained in the full picture into numeric representations known as wavelets. Therefore, it is prone to quality issues arising from noise, occlusion, and variation of the viewpoint. To rectify these shortcomings, researchers turned from the full-picture information to cuboids of spatial-temporal shapes in the foreground. Some [9] have attempted to extract spatial-temporal features, shape structures, shape orientation, and salient outlines of the shape by stacking up silhouettes frame-by-frame, but this was found to be quite inefficient. Researchers improved the shaped-based matching mechanism by using region matching and extracting certain regions [10]. Keeping the appearance of the 3D objects or the region consistent is difficult. Other researchers suggested a flow-based feature model [11] and a spatial-temporal interest point detector model [12], which only processes the flow and points of interest in the local region, respectively.

Local features are obtained relatively directly from tracking the pose and coordinates of the body. Different from remote features, local features are more focused on the movements of the interest points without regard to the background or the full picture. To represent local features, spatial information is often refined from the raw tracking data (e.g., GPS coordinates) using spatial estimation techniques, such as Boolean features [13], polar coordinate features [14], and geometric relative features [15,16], to effectively preserve the spatial information that discriminates different bodily activities. Although these local features are obtained almost directly from the sensing data, many variants derived from different transformation techniques have been proposed by researchers in the hope of better capturing the essence of the spatial information in relation to activity recognition. Refining and abstracting techniques with the aim of generating better local descriptors include, but are not limited to, wireframe or shape-based features [17,18,19].

Recently, indirect methods of feature transformation have gained popularity for their simplicity and efficacy in representing movement and hence activities. One such method is histogram of oriented gradient (HOG) descriptors generation [20], in which the features are generated based on normalised local histograms of image gradient orientations. A variant of HOG, PCA-HOG, in which the HOG features are projected onto the linear subspace by principal component analysis (PCA), thereby reducing the total feature space, has been proposed [21]. Other statistical feature methods exploit the different distributions of statistical features of the data including its mean, variance, deviations, skewness, kurtosis, entropy correlation, and first-order derivatives. Some of these methods have been used and showed improved efficacy [7,22]. Intuitively, it is easy to understand that variance in the sensor data is an apparent result of drastic activities like jumping, dancing and running compared to walking and standing. High opposite correlation does exist at the vertical axis of the sensor data from one leg to another when subjects are walking on stairs or biking.

However, for this paper, we argue that statistical features and wavelet features are not always the most appropriate for building classification models for HAR. As mentioned above, this is because they require repeatedly reloading the full dataset into the supervised learning model, which may lead to time lags during the HAR process. This is especially true when incremental learning is not in use. Consequently, an adaptive method that focuses on local sliding window information should be used. The drawbacks of the current feature transformation methods are the impetus for the new method we propose here, namely, the shadow feature method.

## 3. Proposed Shadow Feature Classification Model

A new classification model is described in this section. It extends the traditional HAR system model in which the raw activity data is pre-processed prior to either training or testing the classifier.

### New Classification Model

**Figure 2:**
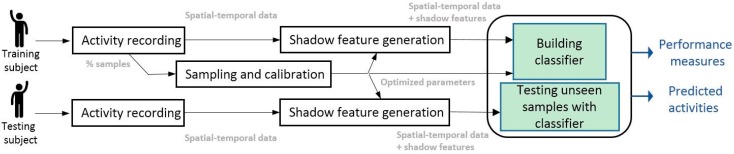
Proposed HAR classification model.

Figure 2 presents an overview of the HAR system embracing our new shadow feature generation method.

The new classification model comprises an activity recording module, sampling and calibration modules, the shadow feature generation module, and a classification module. The activity recording module and classification module are generic. They can be implemented by any suitable software/hardware and algorithms. The recorder can be a camera or sensor of any make, as long as they can capture the activities and produce the corresponding activity dataset as time-stamped ordered sequences. The general format of the activity dataset is a matrix with rows of ordered instances (e.g., each row contains information about the video frame per sampling interval) and columns of attributes or features that describe the information. The training dataset has a labelled column that already contains the types of activity corresponding to each particular row of instance. The training dataset is used to train or build a classifier after passing through the shadow feature generation process that extends the original feature set of the training data with shadow features. In the testing phase, an unlabelled dataset is subject to a shadow feature generation process configured by the same parameters that controlled the shadow feature generation process for the training dataset. Then, the extended testing dataset with the corresponding shadow features is tested against the built classifier, for activity recognition and the predicted result is then obtained from the classifier.

In incremental learning, which is also known as data stream mining, the training dataset streams continuously into the classifier construction process. The dataset passes through the shadow feature generation process as through a pipeline, with new features continuously being generated using a sliding window over the input data stream. Likewise, the testing dataset streams into the classifier at any time (though preferably after some warm-up period at the initial stage of classifier training), the corresponding shadow features are added on the fly, and the results are predicted continuously at the end of the pipeline as streaming progresses.

**Figure 3:**
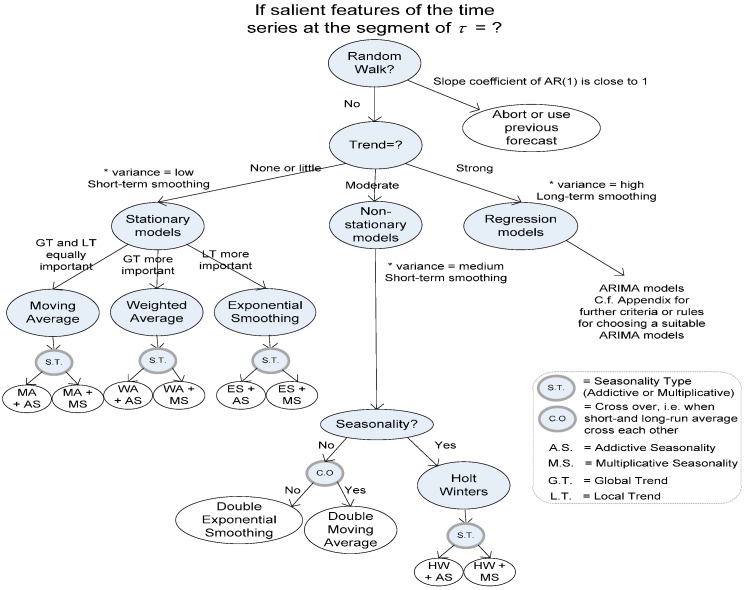
Decision graph for selecting an appropriate curve-fitting method for shadow feature generation.

The sampling and calibration process is an important module in the proposed model. It has two parts. First, small training data samples are taken to evaluate several key parameters of the time series of the training data. The module tests whether the time series is stationary or non-stationary, whether a trend exists and whether the time series is merely random-walk or it can be forecasted. The testing method is detailed in [23]. The decision diagram is reproduced from [23], and shown in Figure 3. Consequently, a suitable time series forecasting method is deduced from the salient characteristic of the time series obtained from the initial samples. The chosen forecasting method will be used as a curve-fitting method for generating shadow features in the subsequent step. The other calibration task is to compute a dynamic Hurst factor, which is used as a decisive factor in estimating the dynamic length of the sliding window. The Hurst factor is related to the fluctuation level and long-term memory dependence.

To keep the proposed model generic, we let the dataset determine which curve-fitting method is used in the shadow feature generation by referring to the decision graph (as in Figure 3) into simple IF-THEN-ELSE rules in a computer program, and the dynamic window size using the Hurst factor. As such, the model is adaptive and generalised for different shapes of time series in HAR.

**Figure 4:**
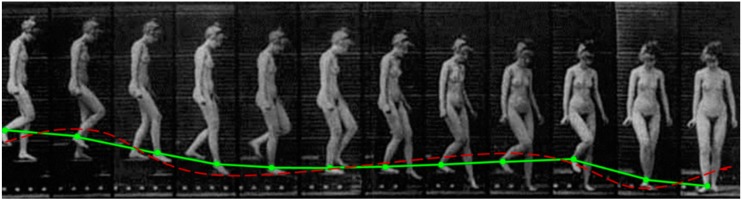
A fitting curve superimposed on a classic cinematographic picture of woman walking down stairs *(Image courtesy of Motion Studies predating Taylorism, Images produced by Etienne-Jules Marey*).

**Figure 5:**
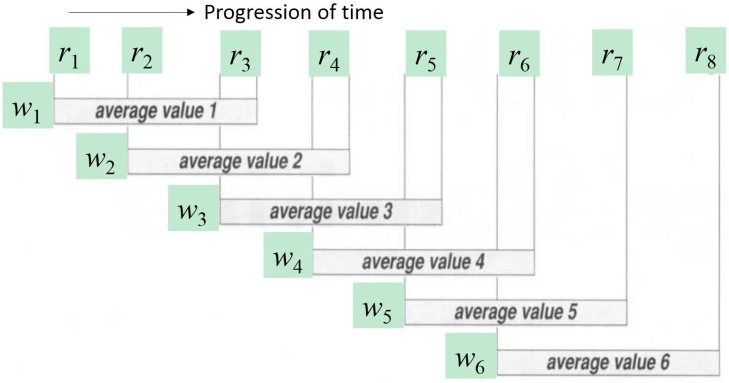
Snapshot of the moving average in action.

In the case of the human activity data to be used in our experiments, such as the one demonstrated in Figure 4, although the time series fluctuate greatly in the time domain, they are stationary and no obvious trend can be observed. The full training set of activity data is visualised in Figure 5. The best fitting curve candidate is the moving average smoothing method. One can recognise, even with the naked eye, that the tri-axial time series have various shapes over a period of sampled time. Each activity that was performed at different times is expressed by the combined tri-axial patterns, each of which has a certain amount of data fluctuation. In Figure 5, sitting and standing have the least amount of fluctuation, while biking, climbing stairs, and walking cause a significant amount of fluctuation. The basic premise of using shadow features is to reinforce the uniqueness of each activity pattern by using its original data fluctuations and their respective curve-fitting patterns, as an extra ingredient in the supervised training of a classifier. In data mining, the shadow features are added as an extra column adjacent to the columns of the original features. The shadow features are created by sliding the window within which the mean across the data points is computed. The length of the sliding window is pegged at the long-term memory dependence approximated by the Hurst factor.

The idea of using additional time series by shadow feature is similar to using lagged variables to incorporate feedback from the dynamic time series over time in econometric forecasting models. The basic lagging variable concept works fine for traditional forecasting where the whole set of data is used in the auto-regression equation. However, for HAR and its dynamic streaming environment, the classifier is continuously refreshed using incremental learning; so a sliding window is adopted and simple time series processing for the shadow feature inference method is preferred and introduced here.

The training/testing dataset takes form of a data matrix, *D_train_*/*D_test_*, with dimensions (*M* + 1) × *N*, where there are *M* + 1 columns covering *M* features and one target class, and *N* rows of data instances. Each column has a time series corresponding to a specific feature. Given a time series *Y_j_* for each original feature *a_j_*, there is a complimentary time series $Y_{j}^{shadow}$ for the corresponding shadow feature $a_{j}^{shadow}$, where *j*∈[1, *M*]. Let *ri* be a data record holding data of *M* features at the *i*th order of *D*. The shadow time series is derived from its counterpart original time series that has the following form:

Y*_j_* = {(*r*_1_, *t*_1_), …, (*r_n_*, *t_n_*)}, *n*∈*N*, and $Y_{j}^{shadow}$ = {($r_{1}^{shadow}$, *t*_1_), …, ($r_{n}^{shadow}$, *t_n_*)}, both time series are synchronized by the same timestamps, where *r_i_* and $r_{i}^{shadow}\;$ share the common data tuple format (*x_i_*_,1_, *x_i_*_,2_, …, *x_i_*_,*M*_|class*_i_*) at the *i*th position of the time series sequence, where *x_i,j_* is a real-number extracted from the raw sensing data with respect to the *j*th feature. Each data tuple *r* in *D_train_* will carry a target class label; and the class is empty in the data records in *D_test_*.

At the beginning of the model building process, a certain amount of initial samples are used for calibration. From there, salient statistics are calculated in the start-up. Hurst factors are computed. The time series in *D_train_*/*D_test_* are determined whether they are stationary or otherwise, as to decide which curve-fitting algorithm we are to use (Figure 3).

For whichever a curve fitting algorithm that is chosen to use, Hurst factors play a part in the shadow feature values generation. Two Hurst exponentials are calculated, one is *H^start^* calculated during calibration from a subset of *Y* (*Y^subset^* ⊆ *Y*), the other is *H^dynamic^* calculated during the curve-fitting process in shadow feature generation. *H^start^* is the standard Hurst exponent defined by the asymptotic behaviour of the standard rescaled range as a function of time period of $Y^{subset}$ that contains certain amount of data points in the initial part of the time series:

(1) $$\varepsilon \left[\frac{ℛ\left(Y^{subset}\right)}{\sigma \left(Y^{subset}\right)}\right]=C{\left(Y^{subset}\right)}^{H^{start}},$$

where ε[·] is the expected value pertaining to the *j*th feature in the bounded time-series $Y^{subset}$, ℛ is the range of the values in the $Y^{subset}$, σ is their standard deviation, and *C* is an arbitrarily chosen constant. The scaled range is calculated as:

(2) $$\frac{ℛ\left(Y^{subset}\right)}{\sigma \left(Y^{subset}\right)}=\frac{max\left(\delta_{1},\;\delta_{2}\ldots \delta_{\left|Y^{subset}\right|}\right)-min\left(\delta_{1},\;\delta_{2}\ldots \delta_{\left|Y^{subset}\right|}\right)}{\sqrt{\frac{1}{\left|Y^{subset}\right|}\sum_{i=1}^{\left|Y^{subset}\right|}{\left(x_{i}-\mu \right)}^{2}}},$$

where μ is the mean, $\mu =\frac{1}{\left|Y^{subset}\right|}\sum_{i=1}^{\left|Y^{subset}\right|}r_{i}$; and $\delta_{t}$ is an element of the cumulative deviate series δ, so $\delta_{t}=\sum_{i=1}^{t}\left(r_{i}-\mu \right)$, for *t* = 1, 2, …,$\;\left|Y^{subset}\right|$. *H^start^* is calculated by fitting the power law in Equation (1) to the initially sampled time series. Then, we repeat the estimation of *H^start^* for each feature *j*. 

At the start, *H^start^* is used as one of the salient indicators [23] in deciding a stochastic model for fitting the time series for shadow feature generation. *H^start^* is also being referred to for quickly finding an appropriate sliding window size. The length of the sliding window is pegged at the long-term memory dependence (LRMD) which is approximated by Hurst factor.

Instead of dealing with a power-like exponential decay and an auto-covariance function, which are heavy in computation, an alternative lightweight approach using Hurst factor is adopted here. Assuming the activity time series is stationary (by which the volunteer was repeating the same movements in the same activity), there should be some minor actions within an overall action, and they do repeat in a similar manner along the way. e.g., jogging, the action can be decomposed lifting up and down, and altering between the pair of legs, recurrently. Similarly for jumping hurdles, where it involves recurrent laps of running for almost the same distance and jumping up at almost regular intervals. Likewise for other sports, e.g., a tennis player moves around within the same court, combined with a kind of periodic raising of the arm and hitting the ball. Quantitative techniques are available to estimate the appropriate window length [24], though they incur substantial computing overheads. A quick alternative is to manually prepare a group of various predefined window lengths according to the durations and contents of the activities. The value of *H^start^* is then used to pick a size by referring to the continuity intensity Table 1 [25]. In general, the stronger the LRMD is, the longer the window size would give a good result. Calculating *H^start^* once at calibration enables us to choose a curve-fitting method and estimating the sliding window length.

During the shadow feature generation process, another type of Hurst factor, called *H^dynamic^*, is calculated along with the curve-fitting process. In moving average smoothing, *H^dynamic^* is a variable whose value is derived from the range of elements within the current sliding window. Then it is updated when the sliding window advances to the next data record. At the same time the window size (tailing position) is dynamically updated according to the latest *H^dynamic^* value.

The *H^dynamic^* is calculated in the same manner as in Equations (1) and (2), except that the data range for *H^dynamic^* is bounded by the data length in the current window *w* along the time series *Y*, instead of $Y^{subset}$. This dynamic Hurst factor $H_{i}^{dynamic}\;$ is calculated iteratively whenever the window advances to the next item, until the end. The Hurst exponent can be drawn on a Log(*R/S*) and Log(*n*) plot; the slope of the regression model is approximated as follows:

(3) $$\mathrm{Log}{\left(R/S\right)}_{w_{i}}=\mathrm{Log}\left(C\right)+H_{i}^{dynamic}\cdot \mathrm{Log}\left(\left|w_{i}\right|\right)\;\forall \;i\in \left[1,N\right],$$

(4) $$H_{i}^{dynamic}=\frac{\mathrm{Log}{\left(R/S\right)}_{w_{i}}-\mathrm{Log}\left(C\right)}{\mathrm{Log}\left(\left|w_{i}\right|\right)}.$$

To illustrate the operation for the example of the moving average, which is being chosen as the curve-fitting method for generating the shadow feature, a snapshot is shown in Figure 5. In this data pre-processing example, a shadow feature is computed by quickly averaging the past |*w*| − 1 data values plus its current data value from the current window frame on *Y*, where *w* = 3 and the window sizes remain the same in this illustration. The computed shadow values are copied to *Y^shadow^*, except for the first |*w*| − 1 data slots. Symbols “?” are inserted instead marking them as unknown values. The window length, *w_i_*, however, may change depending on the latest *H^dynamic^* value, making the shadow feature generation process adaptive to the time series and accommodating the LRMD effect, if any. In reality, of course, large window sizes, e.g., in magnitude of 10, 100, or greater, should be chosen, especially when the data frames are sampled at high frequency by a high-resolution camera.

The same window size values are sent from the pre-processing process to the model induction process (as indicated in Figure 2) for incremental learning in cases where the data stream mining algorithms are in use, optionally. In data stream mining mode, the pre-processing process and model induction process can be synchronized.

Assuming the values of items in the time series characterized by the shadow feature *Y^shadow^* take the same format as *Y*, the item value can be computed rapidly by calculating the mean of the previous *w* data including the current one, as follows:

(5) $$r_{i}^{shadow}=\left(\varphi_{i}+1\right)\cdot \frac{r_{i}+r_{i-1}+\ldots +r_{i-\left(w_{i}-1\right)}}{w_{i}},\forall i\in \left[1,\ldots ,n\right],$$

where $\varphi_{i}$ is a scaling factor signifying the importance or relevance of the data points in the current *i*th window to the shadow feature. The factor can be estimated by normalizing the dynamic Hurst factor, such that:

(6) $$\varphi_{i}=\frac{\left|H_{i}^{dynamic}\right|-0.5}{0.5}.$$

There is an option to assign more weights to the data near the front of the window by multiplying the window positions so that the time series becomes a convolution of data points with different weights. For example, the current movement data has the maximum weight ω, and the second latest has ω − 1 etc., down to one:

(7) $$r_{i}^{shadow}=\left(\varphi_{i}+1\right)\cdot \frac{w_{i}\cdot r_{i}+\left(w_{i}-1\right)\cdot r_{i-1}+\ldots +2r_{\left(i-w_{i}+2\right)}+r_{\left(i-w_{i}+1\right)}}{w_{i}+\left(w_{i}-1\right)+\ldots +2+1},\forall i\in \left[1,...,n\right].$$

Other curve-fitting methods optionally exist, such as the exponential moving average, the exponentially weighted moving average, etc. However, they may not be suitable for fast processing in a HAR incremental learning environment because recursive and power operators are involved, which incur heavy computational expenses.

## 4. Experiments

Two experiments were designed and conducted to verify the efficacy of the proposed classification model with shadow features using two groups of classification algorithms—the traditional batch learning algorithms J48 (CART decision tree), SVM (support vector machine), and ANN (artificial neural network), and incremental learning algorithms typified by HT (Hoeffding Tree), NB (Naïve Bayes), and 1-NN (K-nearest neighbour, *K* = 1) algorithms. The first group classically represents how sensor data are used in batch data mining and the second group represents data stream mining.

### 4.1. Data Sets and Experiment Setups

Two sensor datasets generated from two types of motion sensing device were used for building classification models for HAR. The first dataset, which we term ‘accelerometer’ data, was collected from a smartphone with simple tri-axial spatial information available for public download from the UCI archive (https://archive.ics.uci.edu/ml/datasets/Human+Activity+Recognition+Using+Smartphones). The second dataset, ‘Kinect’ data, collected from a Microsoft Kinect motion sensor, is more sophisticated. accelerometer data represents the use of a wearable unimodal sensor, and Kinect data represents a multimodal motion sensor. The first dataset is generally regarded as wearable sensor data, which are generated locally at the user; the second type represents the sensing data, which are obtained remotely within a short distance. They have wide applications, which have been reported recently, such as [26,27,28] for wearable sensors and [29,30,31] for Kinect-based sensors.

Both data have a target class called ‘Activity’ with information labels that describe what the human activities are with respect to the instances of multi-attribute data. The accelerometer data and Kinect data have total data instances of 496,424 and 81,000 respectively.

**Figure 6:**
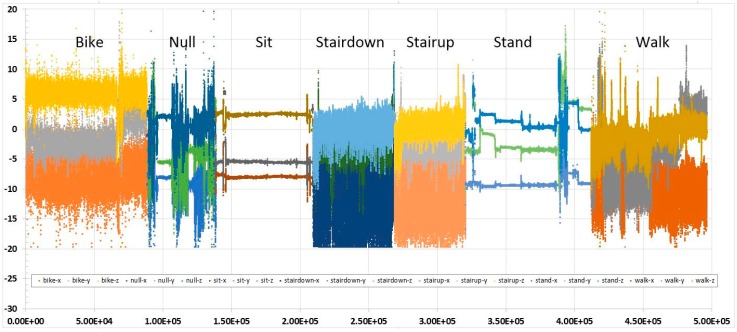
Visualisation of the time series of human activity data by an accelerometer.

However, this accelerometer data has only three attributes/features from the three spatial axes, *x*, *y*, and *z*, that describe the seven human activities of biking (labelled bike), lying down (labelled null), sitting (labelled sit), walking downstairs (labelled stairdown), walking upstairs (labelled stairup), standing (labelled stand), and walking (labelled walk). We visualise the time series of such human activity data as shown in Figure 6. The data were collected from volunteers who were wearing a smartphone (Samsung Galaxy SII) strapped to the waist. Using its embedded accelerometer and gyroscope, three-axial linear acceleration and three-axial angular velocity were captured at a constant rate of 50 Hz by the smartphone. The experiments were video-recorded and data was labelled manually by a human annotator as a post-processing task. This type of data represents a scenario in which simple data streams of minimal spatial attributes are collected; the ratio of attributes to activity types is 3:7. The data volume is more than six times than that of Kinect data. The data archive consists of three pairs of datasets, with each of the three pairs performed by one of the three volunteers. The paired datasets are training datasets with labelled target classes in the last column and testing datasets without labelled information. In our data mining experiment, we used the training dataset collected from the first of the three volunteers for inducing a classification model with and without shadow features. There were two testing modes: (1) *full training*—trying to build a model fitting the original training dataset; and (2) *Train-then-test*—the training dataset comes from one of the three volunteers and after training, the training datasets collected from the other two volunteers are used for testing. The second mode of testing is very challenging because the classification model is trained with data from one person and then used to try to recognise the movements from two different people. The testing dataset combining the training datasets of the two volunteers had a total of 552,151 instances. The actual labels were retained so we would still be able to compute prediction accuracy by comparing the predicted and actual labels.

**Figure 7:**
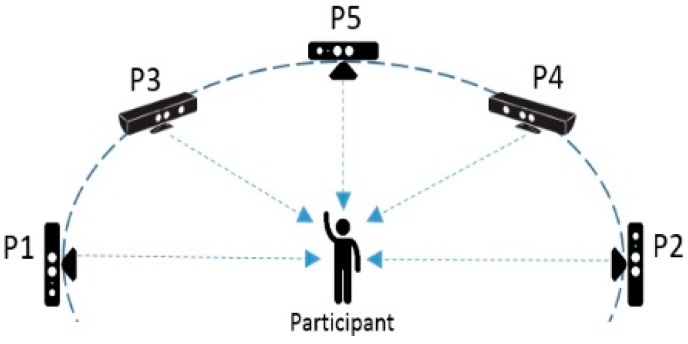
Arrangement of Kinect sensors in our experiment.

The Kinect data is more complex, with 64 attributes that describe 30 different human activities. The attribute data are spatial *x*–*y*–*z* information about various parts of the human body, such as the head, shoulder, elbow, wrist, hand, spine, hip, knee, ankle, and foot. The ratio of attributes to activities is 64:30 (2.13:1). They are downloaded from the Data Repository (https://dlab.sit.kmutt.ac.th/index.php/human-posture-datasets) of the D-lab of King Mongkut’s University of Technology Thonburi, Thailand. A volunteer performed in front of five Kinect cameras placed at different positions doing the 30 postures; the visual postures are coded according to 64 human-to-camera spatial orientations. The data of each position (*x*) was divided into test and train files (P(*x*)testingset.csv and P(*x*)trainingset.csv). The Kinect sensor was placed at five different positions facing the subject at 0, 45, 90, 135, and 180 degrees. P(*x*) stands for position of camera at the *x*th angle of five different degrees used. The five positions of the P(*x*) Kinect sensors are shown in Figure 7. Taking the data of P(5) as the training dataset for supervised learning in a classification model, it is intriguing to observe how the classifier is able to recognise human activities from testing data that are collected from Kinect sensors that are placed in different positions/angles. This arrangement simulates the real-life situation in which the positions of the monitoring cameras during application deployment are likely to be different from those of the camera that was used to register the training dataset from the human participant.

The data streams from accelerometer and Kinect data pose different computational challenges in the context of HAR. Accelerometer data has an attribute-to-activity ratio of <1:2, with few attributes for predicting more activities; and Kinect data has an attribute-to-activity ratio of approximately 2:1, which is the other way around (more attributes predict fewer activities).

In terms of the objective of this paper, which is to investigate the effects of shadow features in data mining, the two datasets offer insights into the effects of the shadow feature methods on large data streams that are generated from different types of sensors with different attribute-to-activity ratios. In addition, two different types of mining algorithm are tested, batch mode and incremental mode. In the context of HAR, the research questions we want to consider are: (1) How effective are the shadow feature methods on sensor data streams that have different data dimensions (scaled by the number of data attributes/data descriptors)? (2) How effective are the shadow feature methods in the data mining modes, batch learning mode, and incremental learning model, with respect to different classification algorithms?

The shadow feature method applied in the experiments is compared to original features only (without any modification on the features) and to the popular Haar wavelet feature transformation method, mainly in testing over the two types of streamed datasets by batch data mining and data stream mining. The common aim of applying the shadow feature methods is to develop an ‘accurate and fast’ classifier. In general, ‘accurate’ means good classification performance refined by a number of performance criteria, such as accuracy, kappa, time consumption in model induction, and F-measure. Accuracy is simply the number of instances that are correctly classified divided by the total number of instances. Kappa is a statistical measure of how generalised the classification model is when different testing datasets are used.

The experiments were run on an Intel Core i7 CPU at 2.2GHz, x64-based processor; the benchmarking data mining software program was WEKA (http://www.cs.waikato.ac.nz/ml/weka/), which is a collection of machine learning algorithms, popular as a benchmarking platform for data mining tasks.

### 4.2. Experiment Results

**Figure 8:**
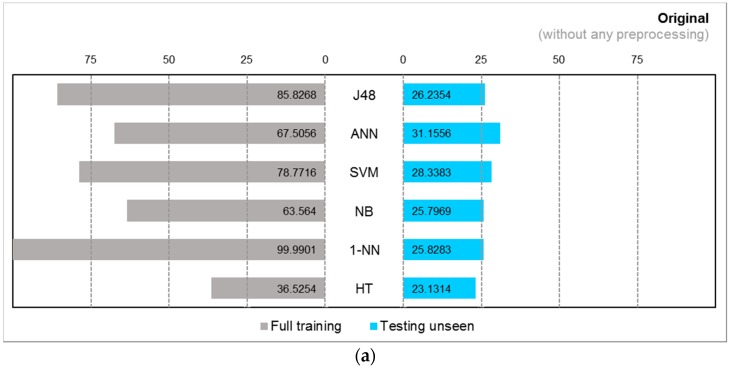

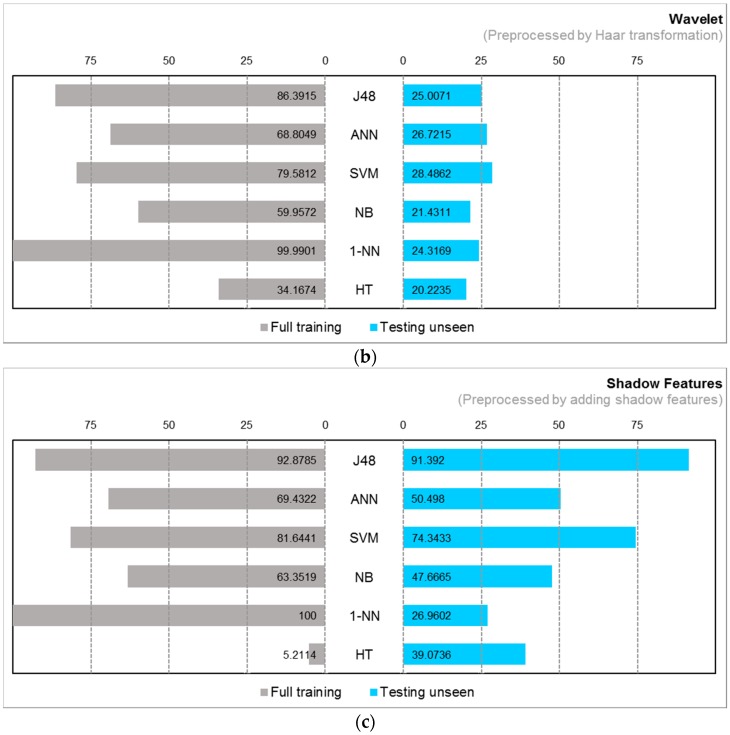
Accuracy of classifiers in accelerometer data with different pre-processing strategies for (**a**) Original; (**b**) Wavelet; and (**c**) Shadow Features.

**Figure 9:**
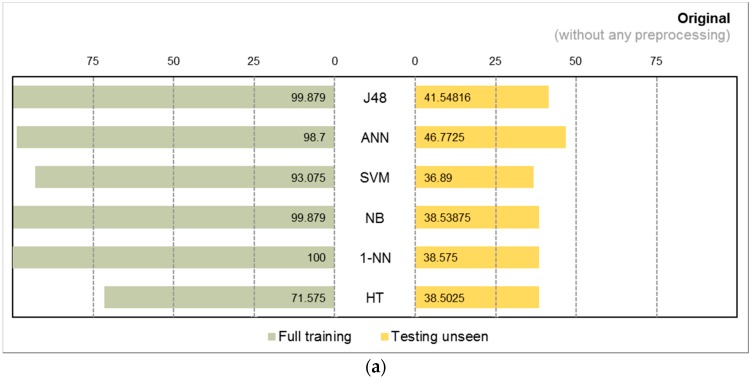

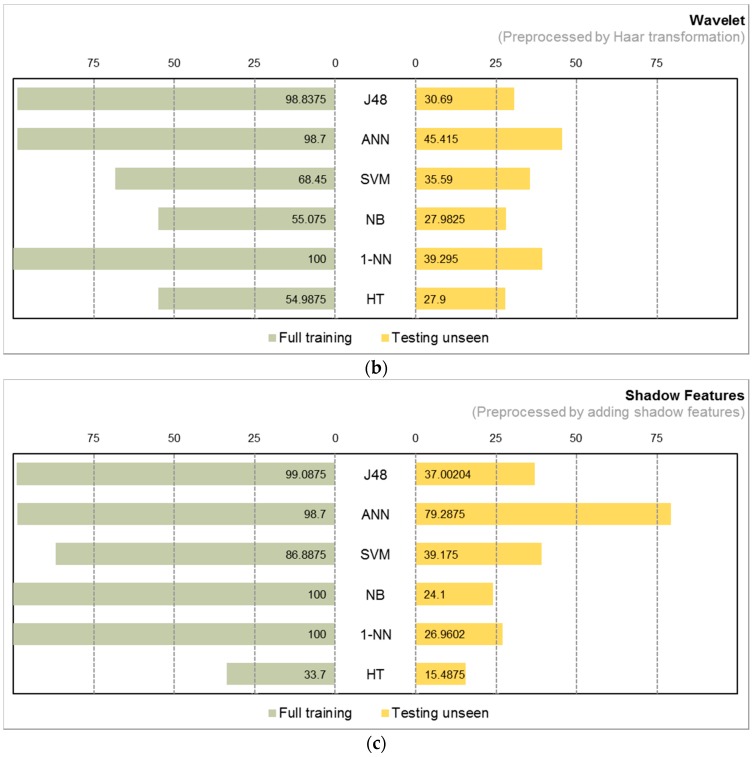
Accuracy of classifiers in Kinect data with different pre-processing strategies for (**a**) Original; (**b**) Wavelet; and (**c**) Shadow Features.

**Figure 10:**
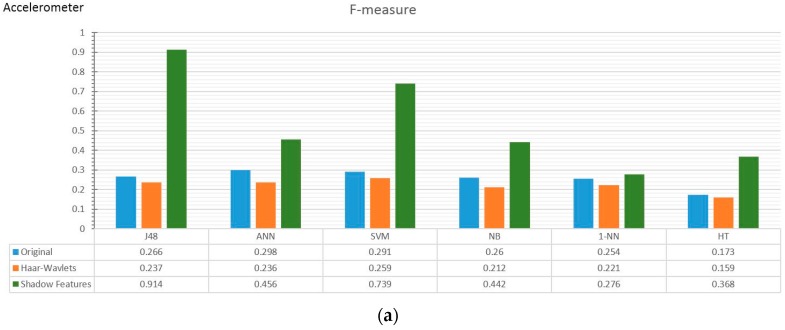

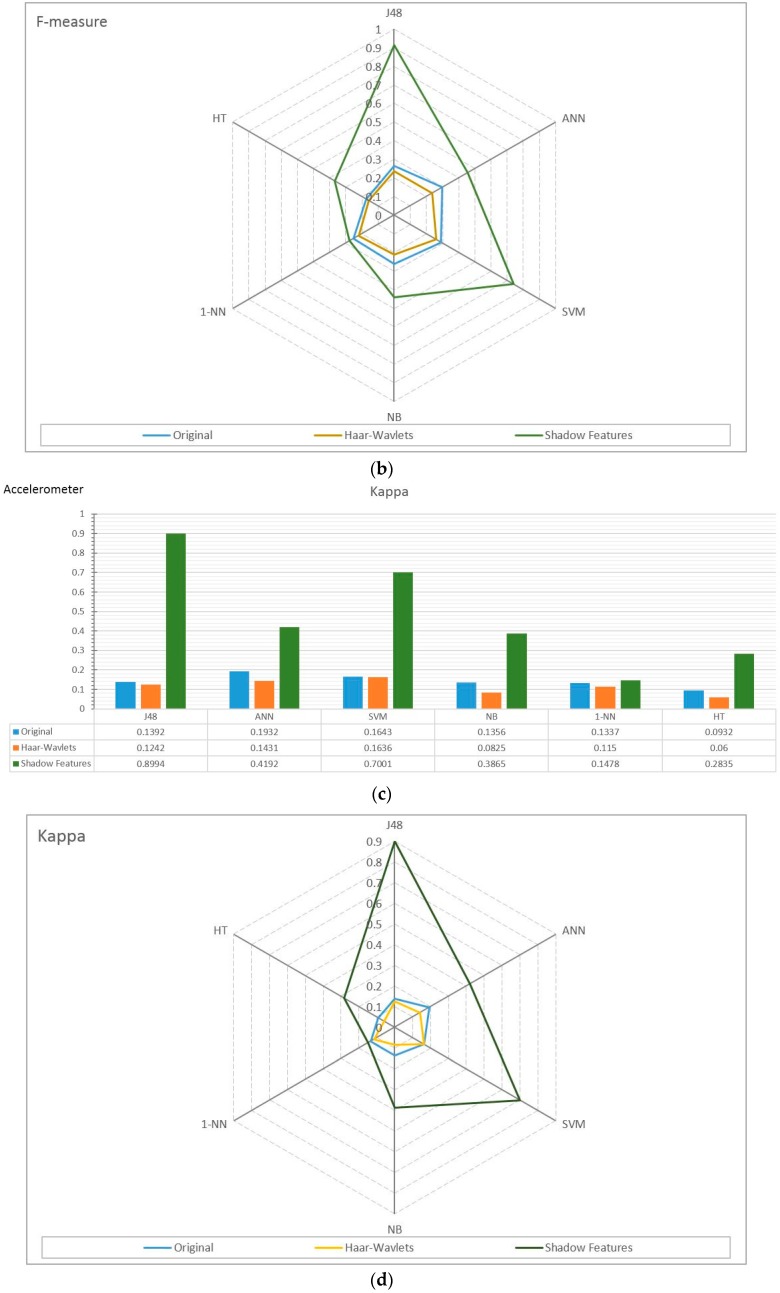

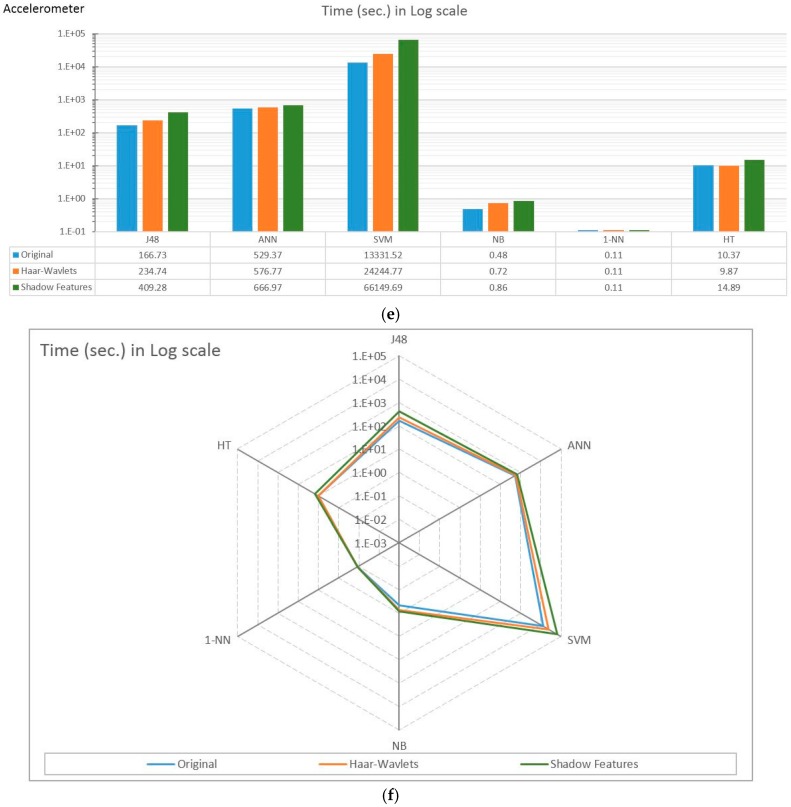
Major performance indicators of classifiers in accelerometer data with different pre-processing strategies based on (**a**,**b**) F-measure of accelerator; (**c**,**d**) Kappa values of accelerator, and (**e**,**f**) Time (sec) in Log scale.

**Figure 11:**
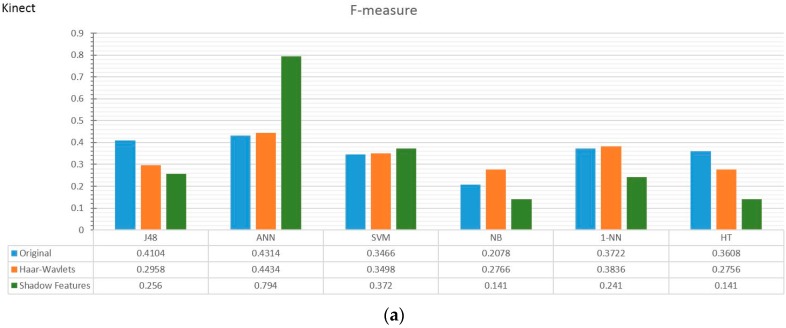

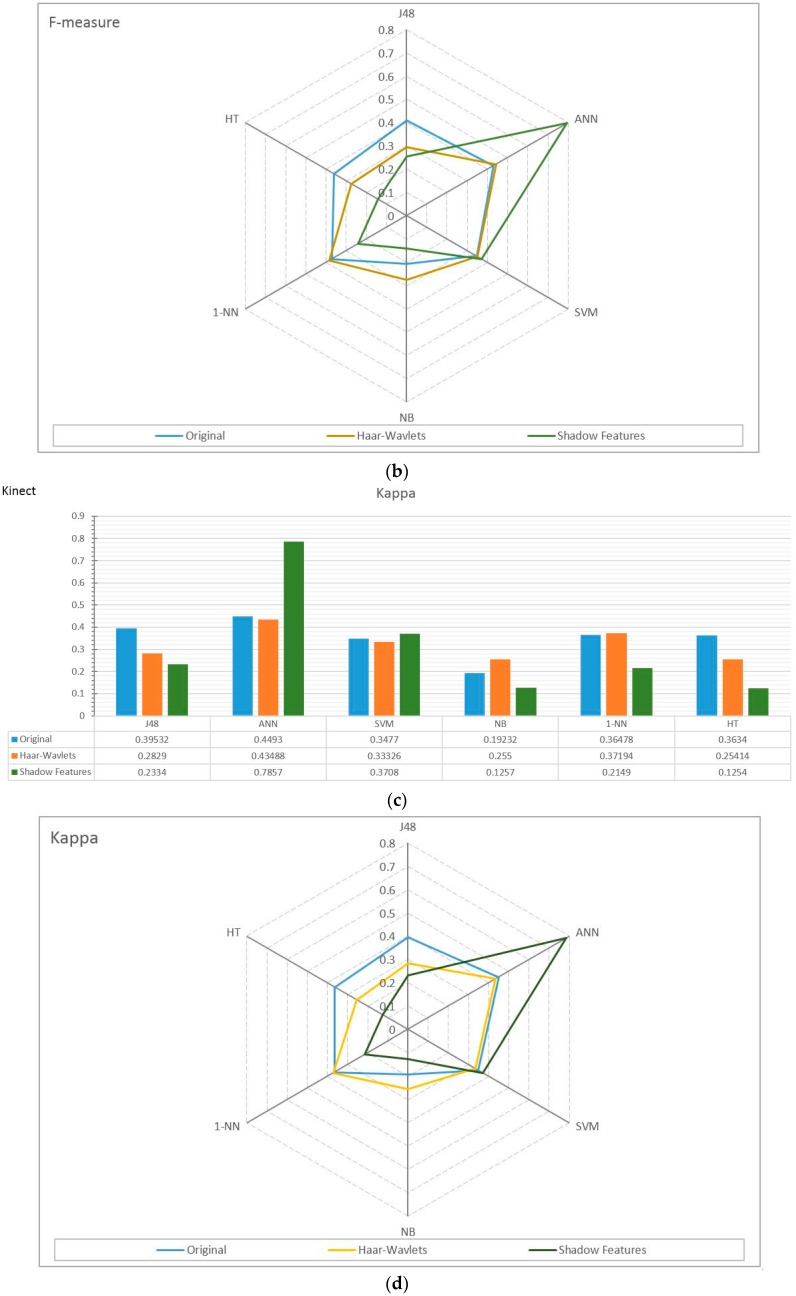

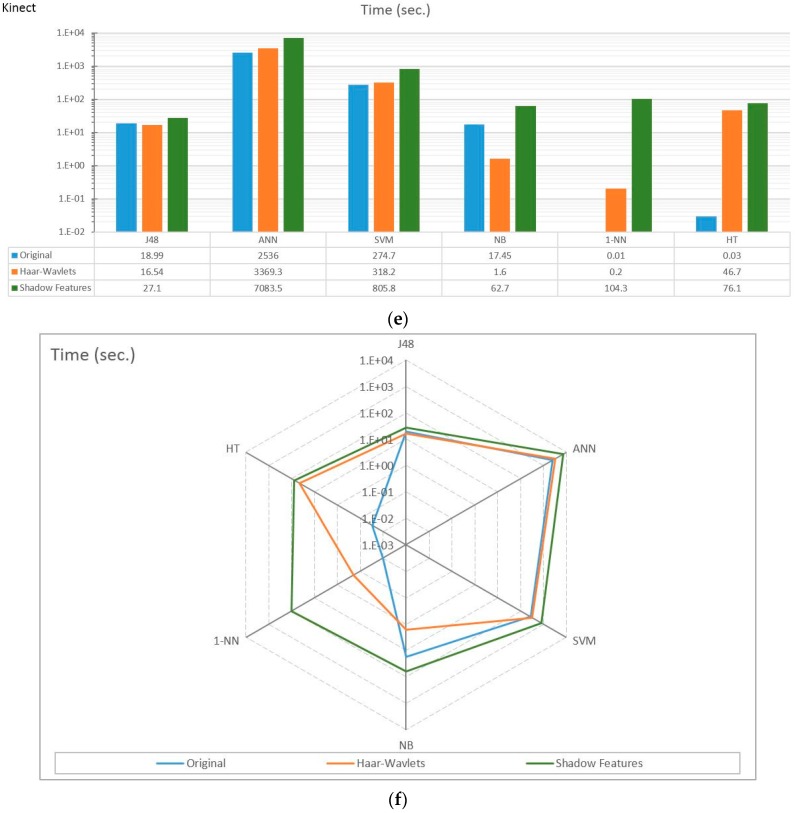
Major performance indicators of classifiers in Kinect data with different pre-processing strategies based on (**a**,**b**) F-measure of accelerator; (**c**,**d**) Kappa values of accelerator, and (**e**,**f**) Time (sec) in Log scale.

Two collections of results from two experiments using different classifiers and different pre-processing strategies are charted: comparisons between full training and train/test, i.e., testing the classifiers with unseen data, and general classification performance in measures of F-measure, kappa, and time taken. The first collection of results done with accelerometer data and Kinect data are shown in butterfly charts in Figure 8a–c and Figure 9a–c, respectively. The second collection of results which are the prominent classification performance indicators are graphed in both bar and radar charts in Figure 10a–f with accelerometer data and Figure 11a–f with Kinect data.

### 4.3. Observations

In the accelerometer data, we observe false high accuracy in the classifiers by full training. In the testing of unseen data, over original data without preprocessing all of the classification algorithms have their accuracies declined to about 25%, except SVM and ANN, which maintained the real accuracies at 28.3% and 31.2%, respectively, as seen in Figure 8 and Figure 9. Even when Haar wavelet transformation was applied, the performance did not improve. The false full training high accuracy is still dominating at a high level and the actual accuracy is still low for most of the algorithms. The group of three traditional classification algorithms barely made it over 25% accuracy, and the incremental learners drop below 25%. Overall, Haar produced worse results than the original without preprocessing. 

However, the shadow feature techniques boosted accuracies throughout the full training and testing unseen. It is apparent that the algorithms benefited from shadow features in Figure 8c where the performance bars far stretched and become more balance than the original and Haar results. In particular, J48 has the greatest gain, achieving full training accuracy at 92.9% and actual accuracy at 91.4%. The ANN has almost doubled its actual accuracies; the SVM increased by more than two-fold; and J48 has increased by more than three-fold. The actual accuracies by all the traditional algorithms exceeded 50%. This shows that shadow features are most effective for traditional learning algorithms, although the incremental learners also improved marginally. 

In the Kinect data, the performances by different preprocessing methods show similar phenomenon in the Kinect data as in the accelerometer data. In general, the accuracies by the original and Haar method exceed 25% for all the algorithms. However, when shadow features are used, the performances of algorithms changed to different degrees. The traditional algorithms all showed remarkable improvements, although the magnitudes of increase are not as great as the accelerometer data. In contrast the incremental learning algorithms shrunk in their performance compared to original and Haar in Kinect data when shadow features are added. 

The F-measure is a balanced performance measure of a test’s accuracy, considering the counts of correct positive results over the counts of all positive results. Figure 10 and Figure 11 show the comparison of different pre-processing strategies and classifiers, in bar chart and radar chart forms, respectively. In the accelerometer experiment, the Haar wavelets method did not show any improvement in F-measure, whereas the shadow feature strategy outperformed the others by multitudes. The extents of improvement are more significant in the traditional group of classifiers, such as J48, ANN, and SVM (at the left side of Figure 10a,c,d), than the incremental learners. In Figure 10c, the kappa statistic is used as performance measure, which is an index that can help people to judge the confidence level of the classification results. It is interpreted as how much the classification power can be generalized to classify other datasets than the one that was used to train the classifier. The kappa index is commonly divided into three levels to evaluate the credibility of classification accuracy. In the top level, the kappa value is ≥0.75, which means that the classification accuracy is high in credibility. A kappa value from ≥0.4 to <0.75 indicates general credibility. Finally, a kappa value of <0.4 indicates a classification accuracy with either low credibility or no incredibility.

The kappa results in our experiment show that the shadow feature strategy can largely improve the credibility and generalizing power of all the classifiers, it especially helps the traditional classifiers move into the creditable range. The kappa of the ANN is over 0.4, for the SVM it is over 0.7, and for J48, a significantly high credibility level close to 0.9, as shown in Figure 10c.

Nevertheless, the traditional classifiers required a very long model training time, especially SVM. Although once a classifier is trained the subsequent testing is computationally fast, a long training time means initial latency and overhead in the HAR before the application can become effectively useful for testing. SVM requires almost three times more time when the shadow feature strategy is used than the original method, making it unsuitable for a HAR environment. Moderate candidates are J48 and ANN. For the group of incremental learners, though they are fast, they are generally underperforming. Shadow features, nonetheless, improved their F-measure performance at a lower time cost compared to traditional classifiers. More future research is encouraged to exploit the prospects of using shadow features in incremental learners.

However, for the Kinect experiment, different effects of shadow features are observed in Figure 11. The shadow features which greatly enhanced the F-measure and kappa in the accelerometer data did not improve the classifiers in the Kinect data, except for ANN and SVM. However, shadow features slowed the model-building times for all of the classifiers in the Kinect data. Even in the traditional classifiers, which are supposed to be able to tap into the advantages provided by the extra shadow features, J48, as a classical decision tree algorithm, decreased in F-measure and kappa. It is a sign of overfitting in the decision tree—it occurs when the machine learning model captures the noise of the data trying to fit the data too well, resulting in an excessively complicated model with low bias and high variance. The major cause of overfitting is a high number of features in the Kinect data; the shadow feature strategy doubled them from 64 to 128, confusing the supervised learning. Furthermore, the two classifiers with the poorest performance with shadow features are NB and HT. They are known to treat features independently. Given that we have so many features, the classifiers are no longer able to recognize the relations of which shadow features that the main features are corresponding to. The training data of 128 features inputted to the learning algorithms appear very random; hence, the low classification efficacy with low bias and high variance. Only the ANN performs very well when empowered by shadow features. The ANN has relatively sophisticated neuro-learning ability, mapping high dimensions of feature values to the activity classes. Shadow features help to boost the F-measure and kappa of the ANN close to 0.8.

## 5. Conclusions

Human activity recognition (HAR) is a challenging machine learning problem because of the dynamic and high-speed sensing data that needs to be processed quickly for inducing a prediction model. In this paper, a novel pre-processing strategy called shadow features generation is proposed with the aim of improving the accuracy of the classifiers. The concept of shadow features is founded based on the observations that bodily movements follow some flows of motions similar to the animation in chronophotography. In addition, spatial information that are fed continuously to training a classification model, shadow features are inferred from them by adding extra dimensions of information in terms of temporal sequence. In the shadow feature strategy, a lightweight mechanism is proposed using simple Hurst exponent and curve fitting functions to infer the secondary features that describe the flows of motion of different body parts for richer information for HAR supervised learning. Two cases of HAR are investigated, one by datasets collected from an accelerometer and the other one by datasets generated from a Kinect camera. The former case represents a training scenario with few attributes but a large amount of data instances. The latter case typifies a skeletal training dataset, which is comprised of many features (tri-axial information from different parts of the model body) and a moderate amount of data instances. Our proposed shadow features are shown by simulation experiments to greatly enhance the classification accuracy of the selected classifiers in the accelerometer data. However, overfitting was observed in the Kinect data when the feature dimensions are doubled by shadow features. SVM and ANN managed to learn well from the Kinect data with shadow features. As future works, new designs of classification algorithms and shadow feature generation algorithms should be studied, so to better harvest the advantages from the shadow features as extra predictors, and to avoid overfitting problems. Especially on algorithmic designs of incremental learning, better induction techniques with consideration of dependency relations between the current features and shadow features warrant further investigation. This research direction is important as HAR is meant to operate in a data streaming environment. For future implementation, combining shadow features with other statistical features, as well as feature selection techniques, should be studied, too. Many possibilities exist in hybridizing shadow features and other techniques, and creating ensemble designs.

## Figures and Tables

**Table 1:** Performance characteristics of Hurst exponent and continuity intensity: Grades 1, −1, fractional Brownian movement; Grades 2, −1, standard Brownian movement; Grades 3, −3, 4, −4, long-term and non-periodic loop (return); Grades 5, −5, reversion.

Scale of Hurst (*H*) Exponent Classifications					
Grade	Range of *H* Exponent	Continuity Intensity	Grade	Range of *H* Exponent	Continuity Intensity
1	0.5 < *H* ≤ 0.55	Very weak	−1	0.45 < *H* ≤ 0.50	Very weak
2	0.55 < *H* ≤ 0.65	Weak	−2	0.35 < *H* ≤ 0.45	Weak
3	0.65 < *H* ≤ 0.75	Normal strong	−3	0.25 < *H* ≤ 0.35	Normal strong
4	0.75 < *H* ≤ 0.80	Strong	−4	0.20 < *H* ≤ 0.25	Strong
5	0.80 < *H* ≤ 1.00	Very strong	−5	0.00 < *H* ≤ 0.20	Very strong
